# Mutant-*RB1* circulating tumor DNA in the blood of unilateral retinoblastoma patients: What happens during enucleation surgery: A pilot study

**DOI:** 10.1371/journal.pone.0271505

**Published:** 2023-02-03

**Authors:** David H. Abramson, Diana L. Mandelker, A. Rose Brannon, Ira J. Dunkel, Ryma Benayed, Michael F. Berger, Maria E. Arcila, Marc Ladanyi, Danielle Novetsky Friedman, Gowtham Jayakumaran, Monica S. Diosdado, Melissa A. Robbins, Dianna Haggag-Lindgren, Neerav Shukla, Michael F. Walsh, Prachi Kothari, Dana W. Y. Tsui, Jasmine H. Francis

**Affiliations:** 1 Department of Surgery, Ophthalmic Oncology Service, Memorial Sloan Kettering Cancer Center, New York, New York, United States of America; 2 Department of Ophthalmology, Weill Cornell Medical Center, New York, New York, United States of America; 3 Department of Pathology, Memorial Sloan Kettering Cancer Center, New York, New York, United States of America; 4 Department of Pediatrics, Memorial Sloan Kettering Cancer Center, New York, New York, United States of America; 5 Department of Pediatrics, Weill Cornell Medical Center, New York, New York, United States of America; 6 Department of Medicine, Memorial Sloan Kettering Cancer Center, New York, New York, United States of America; 7 Department of Pathology and Laboratory Medicine, Weill Cornell Medicine, New York, New York, United States of America; University of Nebraska Medical Center, UNITED STATES

## Abstract

Cell free DNA (cfDNA) and circulating tumor cell free DNA (ctDNA) from blood (plasma) are increasingly being used in oncology for diagnosis, monitoring response, identifying cancer causing mutations and detecting recurrences. Circulating tumor *RB1* DNA (ctDNA) is found in the blood (plasma) of retinoblastoma patients at diagnosis before instituting treatment (naïve). We investigated ctDNA in naïve unilateral patients before enucleation and during enucleation (6 patients/ 8 mutations with specimens collected 5–40 minutes from severing the optic nerve) In our cohort, following transection the optic nerve, ctDNA *RB1* VAF was measurably lower than pre-enucleation levels within five minutes, 50% less within 15 minutes and 90% less by 40 minutes.

## Introduction

Ultrashort fragments of DNA of the retinoblastoma gene are commonly present in the peripheral blood (plasma) of retinoblastoma patients prior to treatment [1–3]. Because the DNA is extracellular it is referred to as “cell free DNA” or **cf**DNA and because the tumor derived cfDNA is from a cancer-causing mutation (*RB1*) it is designated **ct**DNA.

In adult solid cancers, the fates of both cfDNA and ctDNA have been studied following surgery, radiation, chemotherapy and immunotherapy [4–8]. Persistence of ctDNA after any of these treatments is associated with a higher incidence of recurrence and progression of disease [9, 10]. cfDNA increases with lung, kidney and bladder surgery (thought to be related to surgical trauma) and may remain elevated for days to weeks after surgery [11, 12]. ctDNA usually decreases after surgery but elevated levels after surgery suggest ongoing local or metastatic disease. There is little information about the fate of circulating cfDNA during surgery so we explored plasma cfDNA at different time points during enucleation and compared them to pre-enucleation levels in children with advanced unilateral retinoblastoma undergoing enucleation surgery.

## Materials and methods

Plasma ctDNA was analyzed using the MSK-ACCESS liquid biopsy assay with deep sequencing and hybridization capture to detect very low frequency somatic alterations in coding exons from 129 cancer related genes including all exons of *RB1* [1, 3]. This assay was approved by the New York State Department of Health and can detect point mutations (single nucleotide variants/SNV’s), insertions or deletions (Indels), and copy number alterations. Two 10cc Streck tubes of peripheral venous blood were used for each assay. Variant allele frequencies were reported (VAF: the proportion of allele bearing the variants divided by the total number of wild-type plus variant alleles at a given genomic location). Our criteria for a call on MSK-ACCESS for de novo specimens were as follows: only duplex reads are used and 3 (hotspot) or 5 (non-hotspot) are required. For copy number variations (CNV) we use a fold change of -1.5 to call a deletion. Testing was performed using white blood cells (WBC) as a matched normal control which facilitates filtering of germline variants as well as accurate dissemination of mutations associated with clonal hematopoiesis.

Eligible patients for this study were children with unilateral retinoblastoma treated at MSKCC who had measurable *RB1* ctDNA assayed prior to enucleation and also at any timepoint during enucleation. Patients who did not have measurable levels before surgery or did not have blood collected during surgery were not included. Bilateral patients were excluded because the remaining eye could have contributed to the remaining VAF making analysis of the impact of enucleation impossible.

MSKCC Institutional Review Board (IRB) approval was obtained for the study and all parents/guardians signed consent for cfDNA analysis and blood draw.

## Results

A total of 8 *RB1* gene alterations in 6 different patients were identified in this cohort and had VAF’s measured prior to and during enucleation surgery. All enucleated eyes were classified Reese-Ellsworth group Vb and International Classification of Retinoblastoma group E. Table 1 summarizes the mutations detected, patient and tumor characteristics.

**Table 1:** Cell free *RB1* alteration results of unilateral retinoblastoma patients: Pre-enucleation and post-enucleation.

Pt	Age at BL ctDNA (mos)	ctDNA VAF % pre-enuc [95%CI]	Time post-enuc (mins)	ctDNA VAF % post-enuc [95%CI]	ctDNA Somatic RB1 alteration	F/u (mos)	Tumor LBD x ht (mm)	IOP (mmHg)	High risk histopathological features*
3	112	1. 1.63 [1.2–2.2]; 2. 2.0 [1.48–2.69]	5	1. 1.26 [0.9–1.64]; 2. 1.6 [1.24–2.06]	1. RB1 **exon17** p.R556* (c.1666C>T); 2. RB1 **exon8** p.N258Kfs*2 (c.774_786delCAGGAGTGCACGG)	16.6	12 x 19.5	12	massive choroidal & ciliary body invasion; prelaminar optic nerve
5	10	2.92 [2.27–3.75]	20	0.42 [0.22–0.77]	RB1 (NM_000321) **exon17** p.VV516* (c. 1547G>A)	14.9	15 x 13.5	14	none
6	41	1. 2.72 [2.02–3.64]; 2. 3.22 [2.39–4.33]	40	1. 0.2 [.11-.61]; 2. 0.27 [0.11–0.61]	1. RB1 (NM_000321) exon10 p.Q344* (c.1030C>T); 2. RB1 (NM_000321) exon14 p.R445* (c.1333C>T)	19.2	16 x 8	12	none
29	7	3.04 [1.92–4.75]	30	0.2 [0.05–0.64]	RB1 (NM_000321) exon19 splicing variant p.X654_splice (c.1960+1G>A)	3.72	15 x 13	uk	none
30	18	26.25 [24.49–28.10]	30	10.75 [9.57–12.06]	RB1 (NM_000321) exon15 p.R467* (c.1399C>T)	3.68	20 x 6	14	none
31	7	4.88 [4.17–5.69]	12	4.97 [4.18–5.90]	RB1 (NM_000321) exon17 p.W563* (c.1689G>A)	5.0	17 x 8	45	none

BL = baseline, mos = months, ctDNA = circulating tumor DNA, post-enuc = post-enucleation, mins = minutes, VAF = variant allele frequency, 95%CI = 95% confidence interval, LBD = largest basal diameter, ht = height, IOP = intraocular pressure.

**Fig 1:**
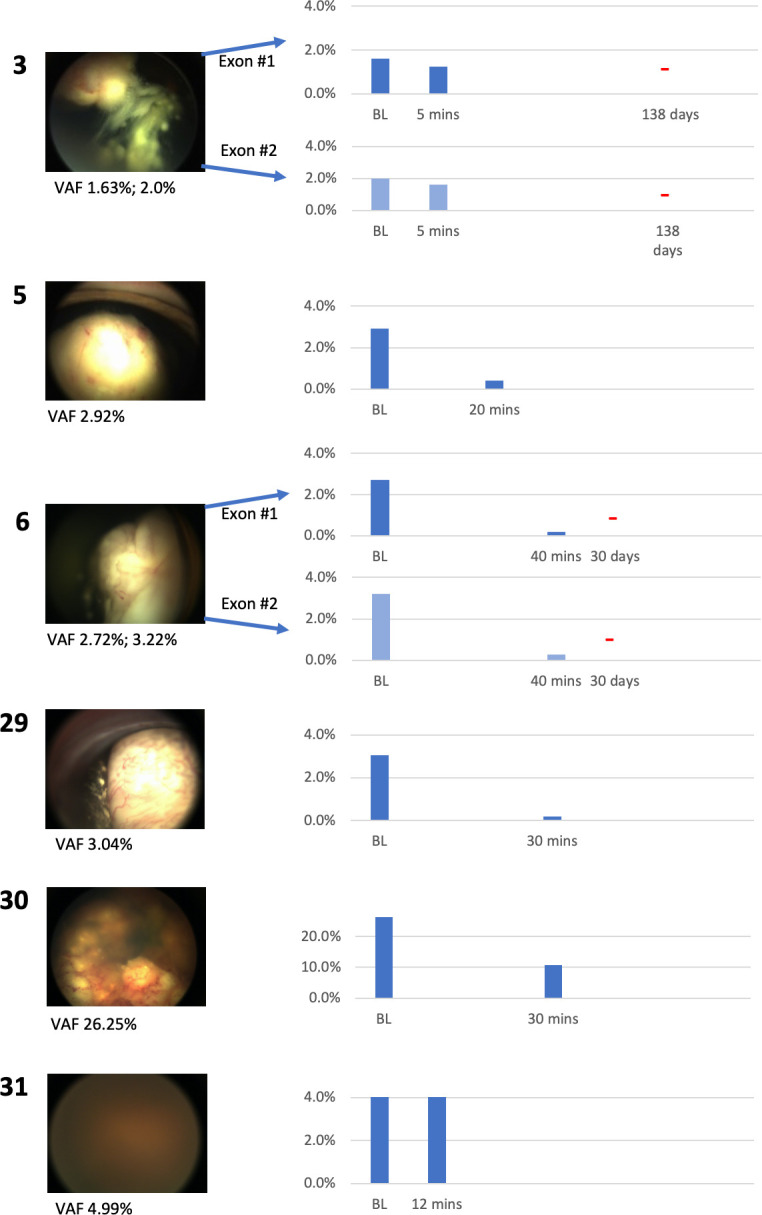
Fundus photographs and clustered bar graphs for four eyes.

The left panels of Fig 1 depict baseline fundus imaging and mutant *RB1* variant allele frequency (VAF) for all eight mutations as labeled by patient number. The right panels show bar graphs for each respective ctDNA *RB1* alteration: the x-axis represents timepoints (BL = baseline, mos = months from baseline), and the y-axis shows plasma circulating tumor *RB1* variant allele frequency percentage. For patients 3 and 6, ctDNA detected two *RB1* alterations shown in separate clustered bar graphs: first exon (dark blue columns) and second exon (lighter blue columns).

**Fig 2:**
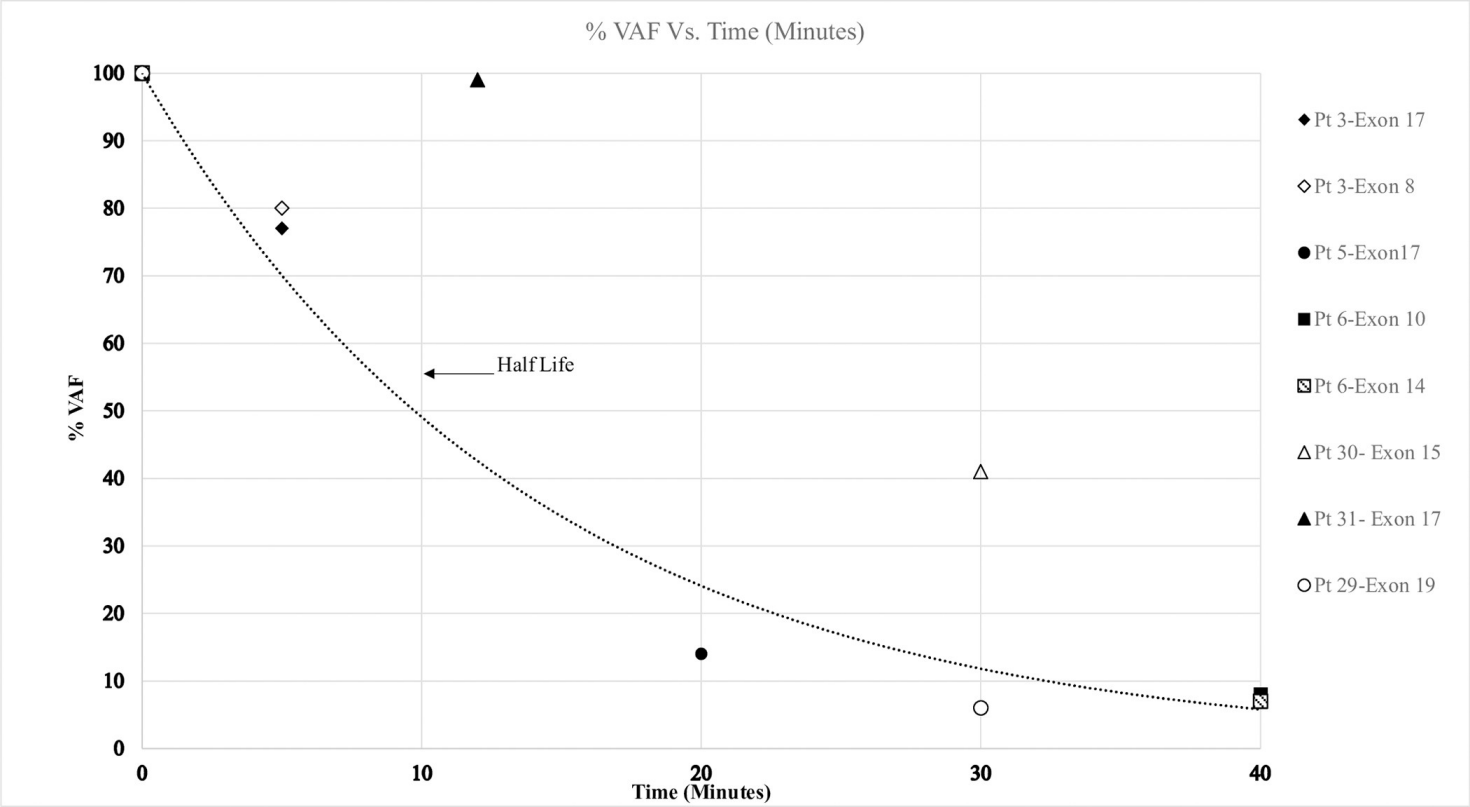
VAF (%) vs. time (minutes).

**Fig 2** demonstrates the normalized plasma levels of ctDNA at different time periods after severing the optic nerve (during the enucleation surgery) for each exon that had been detected prior to the surgery.

## Discussion

Cell free DNA in cancer patients has been reported in blood, saliva, pleural fluid, cerebral spinal fluid, ascites, stool, and urine [11]. It has also been identified in the aqueous humor and blood of retinoblastoma patients at the time of diagnosis [1–3, 13, 14]. Cell free DNA in the plasma of patients with cancer is common and cancer patients have higher levels of cfDNA than non-cancer patients [15]. In a previously reported study from MSKCC on 681 blood samples from 31 solid cancers using MSK-ACCESS, 73% of the samples had structural variants, somatic mutations, and/or copy number alterations [16].

The fate of cfDNA and ctDNA after cancer treatment has been studied. Immediately after surgery in adults for colon, bladder and kidney cancer for example, cfDNA increases for 3–30 days while ctDNA decreases [4, 17]. Persistent elevation of ctDNA is associated with a worse prognosis [15]; the situation with radiation is somewhat different. For example, in lung cancer (non-small cell lung cancer) cfDNA and ctDNA are stable for two hours after radiation and then both increase till the 22nd fraction when it begins to decrease [5]. The pattern with systemic chemotherapy is interesting. In breast cancer, cfDNA decreased after the completion of adjuvant chemotherapy [6]. In ovarian cancer, patients whose ctDNA rose after the first cycle of chemotherapy had improved disease-free survival [18]. The impact of modern immunomodulation on cfDNA in cancer has also been studied. In metastatic melanoma CTLA-4 and PD-1 antibody therapy caused a decrease in ctDNA when measured at 3 weeks and overall survival correlated with this decrease in ctDNA [8]. In lung cancer, immune checkpoint blockage patients with complete disappearance of ctDNA was associated with better outcome than those who had an increase in ctDNA [7].

We recently reported that following intrarterial chemotherapy for intraocular retinoblastoma ctDNA diminishes quickly (90% of patients had none identifiable at 1 month) and in all cases there was no measurable ctDNA in plasma at 3 months [19].

We did not find any elevation of ctDNA after enucleation for unilateral retinoblastoma patients in the current study and all patients but one had decreased plasma levels following severing of the optic nerve.

The half-life of plasma ctDNA in retinoblastoma has not been studied and most of the information about half-life of cfDNA comes from animals without cancer [9, 15, 20–22]. In general, half-life of plasma cfDNA is short (a few minutes to several hours). Although better studied rigorously with multiple time points from the same patient that is impossible in children because (at present) 10–20 cc of blood are needed for each specimen analysis. When we compared post-enucleation *RB1* VAF to pre-enucleation values, the VAF decreased by 13–20% within 5 minutes, 86% by 20 minutes and more than 90% by 40 minutes after transecting the optic nerve. Enucleation did not cause an increase in plasma ctDNA.

The curve depicts baseline and post-transection of the optic nerve *RB1* variant allele frequency (VAF) for three eyes as labeled by patient number. The x-axis represents time in minutes and the y-axis shows plasma circulating tumor *RB1* variant allele frequency percentage. Six patients had eight *RB1* mutations measured prior to enucleation (baseline) and a second measurement within one-hour post-surgery (5 minutes, 12 minutes, 20 minutes, 30 minutes, 40 minutes). In two patients, two distinct mutations were identified and in both cases the decrease in VAF was similar for both exons of the same patient.

Interestingly, it has been shown that cfDNA gets incorporated into the genome of cells and that it can alter the biology of those cells; this phenomenon is thought to influence the subsequent development of metastases in some other cancers [23].

## Conclusions

In this small cohort of naïve unilateral retinoblastoma patients receiving enucleation, the ctDNA *RB1* VAF decreased quickly *during* the enucleation. This decrease was evident within minutes of severing the optic nerve in surgery. By 40 minutes following enucleation, cfDNA VAF had declined to 7–8% of prenucleation VAF. This suggests that the half-life of *RB1* ctDNA after enucleation is short; although this question is best answered by rigorous multiple time point specimen collection from the same patient. These results suggest that detection of ct*RB1* alterations may be a clinically meaningful tool to monitor response to treatment. This also raises the question as to whether post enucleation elevation of cfDNA in blood of unilateral patients after enucleation may indicate ongoing disease outside the eye.
